# Characterization and engineering of the xylose-inducible *xylP* promoter for use in mold fungal species

**DOI:** 10.1016/j.mec.2022.e00214

**Published:** 2022-11-19

**Authors:** Annie Yap, Irene Glarcher, Matthias Misslinger, Hubertus Haas

**Affiliations:** Institute of Molecular Biology, Biocenter, Medical University of Innsbruck, A6020, Innsbruck, Innrain 80-82, Austria

**Keywords:** Fungi, *Aspergillus*, *xylP*, Xylose, Promoter, XlnR, AMM, *Aspergillus*minimal medium, 91bpDS, Duplicated 91bp sequence, Fru, Fructose, Glc, Glucose, p*xylP*, Xylanase promoter, Wt, Wild type, Xyl, Xylose

## Abstract

Conditional promoters allowing both induction and silencing of gene expression are indispensable for basic and applied research. The *xylP* promoter (p*xylP*) from *Penicillium chrysogenum* was demonstrated to function in various mold species including *Aspergillus fumigatus*. p*xylP* allows high induction by xylan or its degradation product xylose with low basal activity in the absence of an inducer. Here we structurally characterized and engineered p*xylP* in *A. fumigatus* to optimize its application. Mutational analysis demonstrated the importance of the putative TATA-box and a pyrimidine-rich region in the core promoter, both copies of a largely duplicated 91-bp sequence (91bpDS), as well as putative binding sites for the transcription factor XlnR and a GATA motif within the 91bpDS. In agreement, p*xylP* activity was found to depend on XlnR, while glucose repression appeared to be indirect. Truncation of the originally used 1643-bp promoter fragment to 725 bp largely preserved the promoter activity and the regulatory pattern. Integration of a third 91bpDS significantly increased promoter activity particularly under low inducer concentrations. Truncation of p*xylP* to 199 bp demonstrated that the upstream region including the 91bpDSs mediates not only inducer-dependent activation but also repression in the absence of inducer. Remarkably, the 1579-bp p*xylP* was found to act bi-bidirectionally with a similar regulatory pattern by driving expression of the upstream-located arabinofuranosidase gene. The latter opens the possibility of dual bidirectional use of p*xylP*. Comparison with a doxycycline-inducible TetOn system revealed a significantly higher dynamic range of p*xylP*. Taken together, this study identified functional elements of p*xylP* and opened new methodological opportunities for its application.

## Highlights

• Mutational analysis identified activating and repressing *xylP* promoter (p*xylP*) elements.
• Reporter analysis revealed bidirectional activity of p*xylP*.
• Promoter engineering improved inducer responsiveness of p*xylP*.
• Comparison with the TetOn promoter indicated superior dynamic range of p*xylP*.

## 1 Introduction

Filamentous fungi are ubiquitously found in nature and are capable of adapting to diverse environments. As they are faced with numerous stressors, fungi represent an important source of natural products exhibiting a wide range of biological activities (Bills and Gloer 2016; Romsdahl and Wang 2019; Keller 2019). The majority of the microbial natural products currently reported in the Natural Products Atlas are derived from fungi (Sorokina et al., 2021). Several of these have been developed into applied drugs with high importance for human health including the first broad-spectrum antibiotic penicillin, hypolipidemic lovastatin and the immunosuppressants cyclosporine and mycophenolic acid (Bills and Gloer 2016; Skellam 2019; Keller 2019). Moreover, filamentous fungi such as *Aspergillus niger* serve as multipurpose cell factory for production of primary metabolites such as citric acid or heterologous proteins (Cairns et al., 2021). For both basic and applied research of these processes, conditional promoters that allow induction and alternatively silencing of gene expression are indispensable, e.g., to functionally characterize essential genes or to manipulate metabolism by modulating gene expression. The *xylP* promoter (p*xylP*) controlling expression of a xylanase from *Penicillium chrysogenum* allows high induction by xylan or its degradation product xylose with low basal activity in the absence of an inducer (Haas et al., 1993; Zadra et al., 2000). p*xylP* was demonstrated to permit conditional gene expression of diverse genes in various mold species including *P. chrysogenum* (Bugeja et al., 2010, 2013; Huber et al., 2019; Janus et al., 2009; Kopke et al., 2013; Pongsunk et al., 2005; Sigl et al. 2010, 2011), *Penicillium marneffei* (Bugeja et al., 2010, 2013; Pongsunk et al., 2005), *Aspergillus nidulans* (Monahan et al., 2006; Wong et al., 2007; Wong et al. 2008; Wong et al. 2009; Tribus et al., 2010; Ma et al., 2018; Pidroni et al., 2018; Wang et al., 2021; Li et al., 2021; Unkles et al., 2014), *Aspergillus fumigatus* (Yasmin et al., 2012; Gsaller et al., 2012; Fazius et al., 2012; Altwasser et al., 2015; Baldin et al. 2015, 2021; Vaknin et al., 2016; Misslinger et al., 2019; Bauer et al., 2019; López-Berges et al., 2021; Handelman et al., 2021; Fabri et al., 2021) and *Sordaria macrospora* (Kopke et al., 2010). *A. fumigatus* is a ubiquitous saprobic fungus but at the same time the most common mold pathogen of humans. This is one of the reasons why it has become an intensively studied model organism (Latgé and Chamilos 2019). Recently p*xylP* was demonstrated to even allow control of *in vivo* gene expression of *A. fumigatus* during murine infection (Bauer et al., 2019). In this invasive aspergillosis model the inducer xylose was supplemented in the drinking water of mice. Consequently, p*xylP* can serve as an alternative to TetOn promoter systems (Helmschrott et al., 2013).

The aim of this study was the characterization of functional elements of p*xylP* in *A. fumigatus* by truncations, deletions and mutagenesis to allow optimization of its application using genes encoding the yellow fluorescent protein mVenus (Kremers et al., 2006) and firefly luciferase (Galiger et al., 2013) as reporters for promoter activity. To exclude genomic position effects, we employed selection marker-free integration of all constructs in single copy at the *fcyB* locus (Birštonas et al., 2020).

## 2 Materials and methods

### 2.1 Fungal strains and growth conditions

All *A. fumigatus* strains in this study were generated in *A. fumigatus* AfS77, which is a derivative of the clinical isolate *A. fumigatus* ATCC 46645 (Hearn and Mackenzie 1980) lacking the *akuA* gene to impair non-homologous end joining (Krappmann et al., 2006; Carvalho et al., 2010). For spore production the strains were grown at 37 °C on *Aspergillus* complex medium (2% (w/v) glucose, 0.2% (w/v) peptone, 0.1% (w/v) yeast extract, 0.1% (w/v) casamino acids, salt solution and iron-free trace elements according to (Pontecorvo et al., 1953).

Plate growth assays were performed by point inoculating 1 x 10^3^ conidia on solid *Aspergillus* minimal medium (AMM) according to (Pontecorvo et al., 1953). The used carbon source is described in the respective experiment. Xylan from oats spelts (SERVA) was used for the characterization of Δ*xlnR* mutants. If not described otherwise, 20 mM glutamine was used as nitrogen source. The plates were incubated for 48 h at 37 °C. All the strains used in this study are listed in Table 1.

**Table 1:** Strains used in this study.

Strain	Genotype	Reference
ATCC 46645	Clinical strain isolated from a human infection (England)	Hearn and Mackenzie (1980)
AfS77	ATCC46645, Δ*akuA::loxP*	(Hartmann et al., 2010; Krappmann et al., 2006)
Δ*xlnR*	AfS77; *ΔxlnR*::*hph*	This study
IG01V, IG02V,	Afs77, Δf*cyB*∷p*xylP* versions driving expression of mVenus	This study
IG03V, IG04V,		
IG05V, IG06V,		
IG13V, IG15V,		
IG16V, IG17V,		
IG20V, IG21V,		
IG22V, IG23V		
IG01L, IG04L,	Afs77, Δ*fcyB*∷p*xylP* versions driving expression of luciferase	This study
IG06L, IG07L,		
IG24L, IG25L		
IG03L*, IG04L*, IG06L*, IG07L* IG24L*	Afs77, Δ*xlnR*::*hph*; Δ*fcyB*∷p*xylP* versions driving expression of luciferase	This study
TetOn^*oliC*^	Afs77, Δ*fcyB*∷tetOn^*olic*^ version driving expression of luciferase	This study

### 2.2 Generation of *A. fumigatus* mutant strains

Oligonucleotides used in this study to introduce the desired genetic manipulation are listed in Table S1. The plasmids containing the p*xylP* truncations and mutations in the reporter constructs were integrated into the *fcyB* locus of *A. fumigatus*, allowing selection for 5-flucytosine resistance without the need of another selection marker (Birštonas et al., 2020). To generate plasmid pIG01V, four DNA fragments were amplified with oligonucleotides shown in Table S1: (i) a plasmid backbone including *fcyB* flanking non-coding regions (NCR) amplified from template p*fcyB* (Birštonas et al., 2020), (ii) p*xylP*, amplified from template pMMHL15 (Misslinger et al., 2018), (iii) codon optimized yellow fluorescent protein derivative Venus encoding sequence (Gsaller et al., 2014) with mutations I152L and A206K to turn Venus into mVenus, and (iv) the *trpC*-terminator sequence, amplified from P*gpdA*_LacZ_AtTrpCTerm_pJET1.2 (Gressler et al., 2011). All fragments were subsequently assembled using NEBuilder® HiFi DNA Assembly (New England Biolabs). For pIG15V, p*xylP* was amplified from pIG01V and integrated in reverse-complementary direction in pIG01V backbone using NEBuilder. Plasmids pIG02V, pIG03V, pIG04V, pIG05V, pIG06V, pIG13V, pIG16V, pIG17V, pIG20V, pIG21V, pIG22V and pIG23V were generate from pIG01V via site directed mutagenesis using Q5® Site-Directed Mutagenesis Kit (New England Biolabs) and primers shown in Table S1. Mutations and deletions in the respective plasmids are shown in Supplementary Figure S1.

To generate the plasmid pIG01L with firefly luciferase as the reporter gene, four fragments were amplified using oligonucleotides shown in Table S1: (i) a plasmid backbone including the *fcyB* NCR, amplified from template p*fcyB* (Birštonas et al., 2020), (ii) p*xylP* fragments, amplified from template pIG01V, (iii) codon optimized *Photinus pyralis* luciferase gene (GenBank accession numberKC677695) (Galiger et al., 2013), and (iv) the *trpC*-terminator sequence amplified from pIG01V. All fragments were subsequently assembled using NEBuilder® HiFi DNA Assembly (New England Biolabs). Plasmids pIG03L, pIG04L, pIG06L, and pIG07L were generated from pIG01L via site directed mutagenesis using Q5® Site-Directed Mutagenesis Kit (New England Biolabs) and primers as shown in Table S1.

To generate pIG24L with both putative XlnR sites mutated, a fragment was generated by PCR using pIG17V as template. The fragment was assembled with the previously amplified plasmid backbone including the *fcyB* NCRs, luciferase and *trpC* terminator gene using NEBuilder® HiFi DNA Assembly (New England Biolabs).

To generate pIG25L containing a third 91bpDS (see below), a synthetic 107 bp DNA fragment with 70% similarity to the other two 91bpDS (generated by Integrated DNA Technologies, Inc, Iowa, USA) was assembled with the previously amplified plasmid backbone including *fcyB* sites, the p*xylP* fragment amplified from template pIG03V, and the previously amplified luciferase and *trpC* terminator using NEBuilder® HiFi DNA Assembly (New England Biolabs). In the third 91bpDS, non-conserved nucleotides between the two original 91bpDS were exchanged to other nucleotides (Supplementary Figure S1B) to avoid homologous recombination with the original 91bpDS due to sequence identity.

To generate the plasmid TetOn^*oliC*^ with firefly luciferase as the reporter gene, four fragments were amplified using oligonucleotides shown in Table S1: (i) a plasmid backbone including the *fcyB* NCR, amplified from template p*fcyB* (Birštonas et al., 2020), (ii), the tetOn^*oliC*^ fragment amplified from template pJW128 (Neubauer et al., 2015), (iii) codon optimized *Photinus pyralis* luciferase gene (GenBank accession numberKC677695) (Galiger et al., 2013), and (iv) the *trpC*-terminator sequence amplified from pIG01V. All fragments were subsequently assembled using NEBuilder® HiFi DNA Assembly (New England Biolabs).

**Fig. 1:**
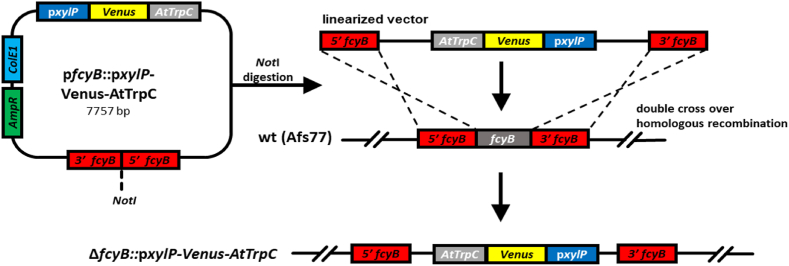
Scheme of genomic insertion of the reporter gene cassettes in *A. fumigatus* by homologous recombination. The generated plasmids were linearized with *NotI* before transformation. The 3′- and 5′-*fcyB* NCR allowed an exchange of the original *fcyB* gene with the plasmid sequence. For simplification, the plasmid regions are not shown in the genomic integration.

After *Not*I or *Pme*I (TetOn^*oliC*^) digestion-mediated linearization, all reporter constructs were integrated in single copy at the *fcyB* locus (Fig. 1), which allows selection for resistance to 5-flucytosine and evades the necessity for a heterologous selection marker (Birštonas et al., 2020).

The XlnR lacking mutant was generated by replacement of the *xlnR* coding region by the hygromycin resistance cassette (*hph*) via homologous recombination according to (Fraczek et al., 2013). Therefore, the 5′-NCR of *xlnR*, the hygromycin resistance cassette (*hph*) and the 3′-NCR of *xlnR* were amplified by PCR with oligonucleotides mentioned in Table S1, using genomic DNA as a template for the NCRs and the plasmid pMMHL69 for *hph*. Subsequently, the three fragments were then linked together via fusion PCR using the nested primers.

Plasmids and PCR products were purified (Monarch PCR and DNA Cleanup Kit, New England Biolabs) and used for transformation in *A. fumigatus*. The transformation of *A. fumigatus* AfS77 was performed according to (Tilburn et al., 1983). Selection of transformants was carried out on AMM plates with 0.2 mg/mL hygromycin B, or 10 μg/mL flucytosine (TCI©, Eschborn, Germany). Correct genetic manipulations were proven by Southern blot analysis (Supplementary Figures S2–S5) and growth assays.

### 2.3 *In vivo* determination of promoter activity

Promoter activities were assessed by measuring mVenus fluorescence intensity in 96-well microtiter plates (Nunc™). Each well contained 0.1 ml AMM inoculated with 10^4^ spores. Plates were incubated for 18 h at 37 °C. Subsequently, absorbance and fluorescence signals were quantified using a CLARIOstar Plus® microplate reader (BMG LABTECH). Absorbance was measured at 280 nm, spiral well scan. For detection of mVenus fluorescence, 497-20 nm excitation and 540-20 nm emission was used. Each reporter strain was analyzed in biological triplicates followed by subtraction of background fluorescence recorded from untransformed recipient strain (wt).

For measuring the bioluminescence of luciferase reporter strains*,* LUMITRAC 96-well plates (Greiner Bio-ONE) were used. Spores were inoculated in AMM to obtain a final concentration of 1.5 × 10^4^ spores in 0.1 ml. After incubation for 18 h at 37 °C, 20 μL of 0.6 mM D-luciferin (Synchem UG & Co.KG, Felsberg/Altenburg, Germany) in PBS was added. The bioluminescence was recorded at 580-80 nm using spiral well scan employing a CLARIOstar Plus® microplate reader (BMG LABTECH). Each reporter strain was analyzed in three biological triplicates followed by subtraction of background luminescence recorded from untransformed wt strain.

## 3 Results

### 3.1 In silico analysis reveals several putative regulatory sequences in p*xylP*

**Fig. 2:**
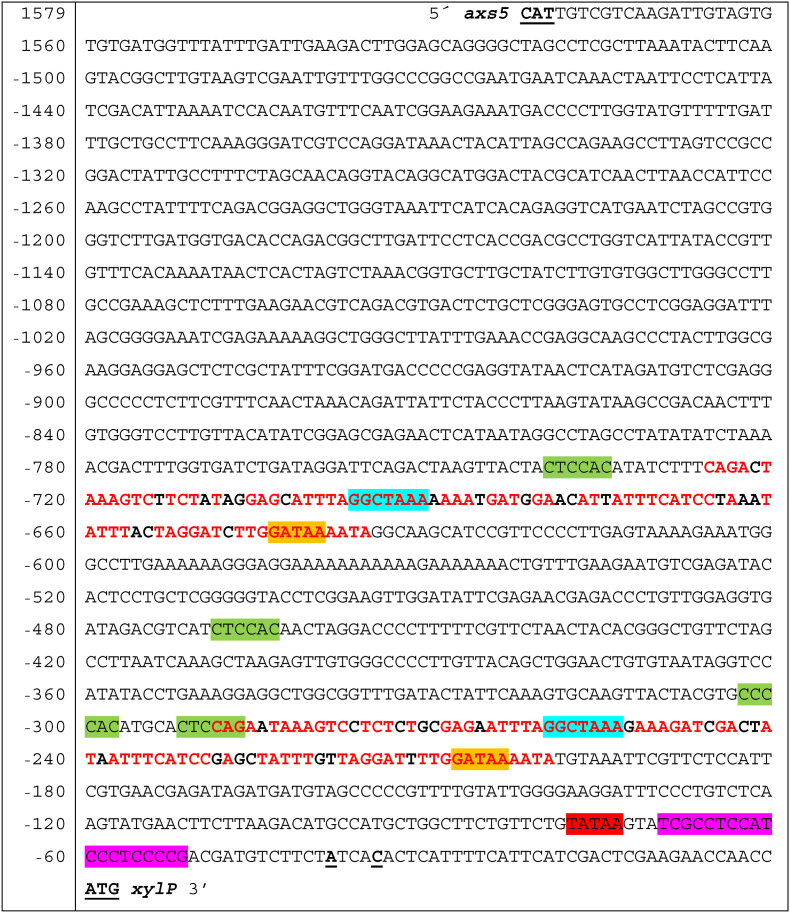
Nucleotide sequence of the bidirectional promoter driving *xylP* and *axs5*. The translation start sites of *xylP* and *axs5* and the transcription start sites of *xylP* are in bold and underlined. The duplicated 91bpDS region is shown in bold letters with conserved nt in red. Putative functional sequences are shadded: 5′-TATAA-3′ in red, pyrimidine-rich sequence 5′-TCGCCTCCATCCCTCCCCG-3′ in pink, 5′-GATAA-3′ within 91bpDS in orange, putative XlnR binding sites (5′-GGCTAAA-3′) in blue, and CreA motifs (5′-SYGGRG-3′) in green. nt numbering refers to the *xylP* translation start codon. (For interpretation of the references to colour in this figure legend, the reader is referred to the Web version of this article.)

The p*xylP* nucleotide sequence is displayed in Fig. 2 (Zadra et al., 2000). A blastx search (https://blast.ncbi.nlm.nih.gov/Blast.cgi) indicated that the 5′-upstream 64 bp of the originally used 1643-bp p*xylP* sequence encodes the N-terminus of the alpha-L-arabinofuranosidase Axs5 (Sakamoto et al., 2011), which suggests that this promoter functions bi-directionally. For the initial use of p*xylP* about 20 years ago, the translation start region 5′-caacATGa-3’ (translation start codon in capital letters) was mutated to 5′-caaccATGg-3’ (*NcoI* recognition site underlined) to allow digestion with the restriction enzyme *NcoI* because recombinant cloning strategies resided on restriction enzyme-mediated genetic engineering at that time. This p*xylP* variation was kept throughout the current study. As previously reported (Zadra et al., 2000) and shown in Fig. 2, p*xylP* contains a largely duplicated 91-bp sequence, here termed 91bpDS, displaying 80% sequence identity. The duplication of this sequence might indicate that it contains important regulatory sequences. Both 91bpDS copies contain a perfectly conserved putative XlnR binding motif and a perfectly conserved GATAA motif. The xylose-induced Gal4-type transcription factor XlnR has been shown to act as transcriptional activator of the xylanolytic system in *A. niger, A. oryzae* and *A. nidulans* (van Peij et al., 1998; Tamayo et al., 2008; de Souza et al., 2013; Kowalczyk et al., 2014). GATAA motifs are recognized by so called GATA-type transcription factors. *Aspergillus* species possess six GATA-type transcription factors termed AreA, AreB, SreA, LreA, LreB and NsdD. AreA and AreB mediate regulation of nitrogen and carbon metabolism (Chudzicka-Ormaniec et al., 2019; Haas et al., 1997), SreA controls iron acquisition (Oberegger et al., 2001; Schrettl et al., 2008), LreA and LreB allow light response (Purschwitz et al., 2008) and NsdD coordinates sexual and asexual development (Lee et al., 2014). Moreover, p*xylP* contains five putative CreA binding sites outside of the 91bpDS copies. The Cys_2_His_2_-type transcription factor CreA mediates carbon catabolite repression, which ensures that genes for the degradation of less preferred carbohydrates such as xylan are turned off in the presence of favorable carbon sources like glucose for economization (Kowalczyk et al., 2014). A putative TATA box is found 34 bp upstream of the most distal transcription start site identified (Haas et al., 1993); this motif might be recognized by the TATA-binding protein (TBP), which is the most conserved general transcription initiation factor (Kramm et al., 2019). Between the putative TATA box and the transcription start site, a pyrimidine-rich sequence is present. Transcriptome analysis in *A. nidulans* revealed an enrichment in pyrimidines immediately upstream of the first transcription start site (Haas et al., 1993; Sibthorp et al., 2013), which might be important to determine the transcription start site and the efficiency of transcription (Kinghorn and Turner 1992; Ballance 1986).

### 3.2 Promoter studies employing mVenus as reporter enabled identification of functional sequences in p*xylP*

**Fig. 3:**
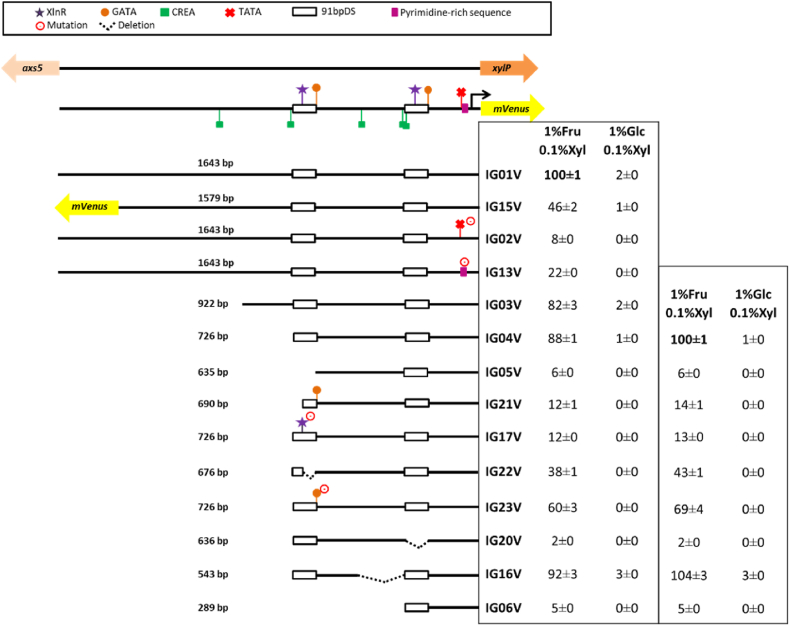
**Truncation, deletion and mutation studies identified functional sequences in p*xylP* using mVenus as reporter for promoter activity**. Promoter activity was measured as described in Material and Methods. Shown values are the mean ± STD of biological triplicates normalized to either IG01V (left) or IG04V (right) grown with 1%Fru/0.1%Xyl; original data are shown in Supplementary Table S2B. Strains were grown for 18 h at 37 °C. Values of growth with 1%Glc as carbon source are not shown as no promoter activity was detected under this condition for any of the promoter constructs.

In order to identify functional elements in p*xylP,* the originally used promoter fragment and 13 versions with truncations, deletions or mutations were fused to the gene encoding yellow fluorescent protein mVenus (Kremers et al., 2006; Kodama and Hu 2010) as reporter for promoter activity (Fig. 3). To exclude influences from the integration locus in the *A. fumigatus* genome, all constructs were integrated in single copy at the *fcyB* locus (Fig. 1), which allows selection for resistance to 5-flucytosine and evades the necessity for a heterologous selection marker (Birštonas et al., 2020). For *in vivo* quantification of promoter activity, fungal strains were grown in minimal medium with different carbon sources in 96-well microtiter plates for 18 h at 37 °C (Table 2). As previously reported (Zadra et al., 2000), the originally used 1643-bp p*xylP* (IG01V) showed high activity with 1% xylose (1%Xyl) as carbon source, while no activity was detectable with 1% glucose (1%Glc) or 1% fructose (1%Fru) as sole carbon sources. Combination of 1%Glc with 0.1, 0.5 or 1%Xyl increased the promoter activity to 2, 21 and 33%, respectively. In 1%Fru combined with 0.1%Xyl (1%Fru/0.1%Xyl) p*xylP* activity reached 91% of that with 1%Xyl. Taken together, these data underline the repressive effect of glucose on p*xylP* activity in the presence of the inducer xylose and define fructose as a non-inducing and largely non-repressing carbon source. Therefore, in the following assays promoter activity of the p*xylP* versions was analyzed in 1%Fru/0.1%Xyl (non-repressing/inducing condition), 1%Glc/0.1%Xyl (repressing/inducing condition) and 1%Glc (repressing/non-inducing condition) to ensure similar availability of carbon source and inducer.

**Table 2:** **Xylose-mediated induction of p*xylP* is repressed by glucose but not fructose.** Promoter activity of IG01V was measured as described in Material and Methods. Shown values are the mean of biological triplicates normalized to 1%Xyl ± STD; original data are shown in Supplementary Table S2A.

	Carbon source						
	1%Glc	1%Glc 0.1%Xyl	1%Glc 0.5%Xyl	1%Glc 1%Xyl	1%Xyl	1%Fru	1%Fru 0.1%Xyl
Promoter activity [%]	0 ± 0	2 ± 0	21 ± 1	33 ± 0	**100 ± 9**	0 ± 0	91 ± 2

To simplify comparison, the activity of all promoter versions was normalized to that of IG01V or IG04V, respectively (Fig. 3). The inverted 1579-bp p*xylP* construct driving expression of *axs5* (IG15V) showed 46% of the activity of IG01V with 1%Fru/0.1%Xyl, which underlines that p*xylP* indeed functions bidirectionally and indicates that the arabinofuranosidase-encoding *axs5* gene shows lower expression compared to the xylanase-encoding *xylP* gene. Mutation of the 5′-TATAAG-3′ sequence 34 bp upstream of the most distal transcription start site to 5′-GGATCC-3′ caused a dramatic drop of promoter activity to 8% under non-repressing/inducing conditions indicating that this sequence might indeed be the TATA box. Replacement of the pyrimidine-rich sequence 5′-TCGCCTCCATCCCTCCCCG-3′ downstream of the putative TATA box by the Tet operator sequence 5′-TCCCTATCAGTGATAGAGA-3’ (Helmschrott et al., 2013) in IG13V, which reduces the pyrimidine content in this region from 84% to 47%, decreased the promoter activity to 22%. Truncation of p*xylP* to 922 bp in IG03V or to 725 in IG04V, which eliminates one and two of the putative CreA sites, respectively, retained 82% and 88% of the promoter activity in 1%Fru/0.1%Xyl. These data indicate that the major regulatory elements are contained within the 725-bp p*xylP* fragment but that the upstream region contains further activating elements.

As all further promoter manipulations were conducted in IG04V, their promoter activity was normalized to that IG04V (Fig. 3). Truncation of the distal 91bpDS containing a putative XlnR recognition motif and a 5′-GATAA-3′motif in IG05V caused a dramatic decrease of activity to 6% in 1%Fru/0.1%Xyl, which emphasizes the importance of this duplicated region. Elimination of the putative XlnR motif in the distal 91bpDS by either truncation of p*xylP* to 690 bp (about the 5′-half of 91bpDS) in IG21V or replacement of the putative XlnR-recognition motif 5′-GGCTAAA-3′ by 5′-CATTAAA-3′ in IG17V decreased the promoter activity to 14% and 13%, respectively, compared to that of IG04V. These results strongly indicated the importance of XlnR for activation of p*xylP*. The higher activity of these promoter versions compared to lack of the entire distal 91bpDS in IG05V (14%/13% versus 6%) indicated additional regulatory elements in the distal 91bpDS element. In agreement, deletion of the 3′-half of the distal 91bpDS in IG22V, which conserves the XlnR binding motif, decreased the promoter activity to 43% compared to IG04V. Furthermore, mutation of the 5′-GGATAA-3′ sequence to 5′-GTCGAA-3′ in IG23V reduced the promoter activity to 69% compared to IG04V, which indicates a role of a GATA-factor in activation of p*xylP*. Comparison of promoter activity of IG22V and IG23V (43% versus 69% compared to IG04V) might indicate additional regulatory elements apart from the identified GATAA motif in the 3′-half of the distal 91bpDS. Deletion of the proximal 91bpDS in IG20V decreased the promoter activity to 2% compared to IG04V. Together with the decreased promoter activity caused by deletion of the distal 91bpDS in IG05V, these data indicate that both copies of 91bpDS are required for full activity of p*xylP*.

Notably, repression by glucose during growth with 1%Glc/0.1%Xyl was largely retained in all investigated promoter versions, even in IG16V, which lacks all predicted CreA motifs (Fig. 3). These results might indicate that p*xylP* is not subject to direct carbon catabolite repression. Moreover, neither of the analyzed p*xylP* versions displayed any detectable promoter activity under repressing/non-inducing conditions, i.e., with 1%Glc (data not shown).

### 3.3 Identification of *A. fumigatus* XlnR

**Fig. 4:**
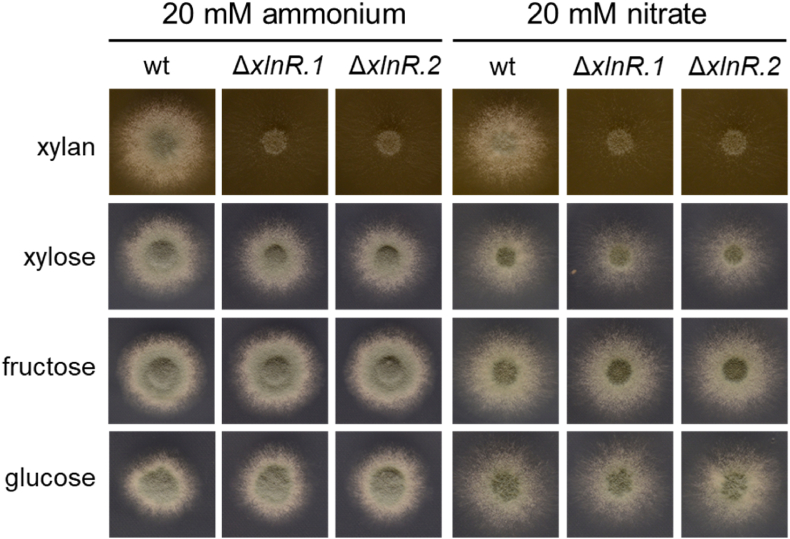
**Deletion of the XlnR-encoding gene causes a strong growth defect of *A. fumigatus* on xylan, a slight growth defect on xylose but no growth defect on glucose or fructose as carbon source.** 10^3^*A. fumigatus* conidia were point-inoculated on solid AMM containing 1% of the indicated carbon source and either 20 mM ammonium or 20 mM nitrate as nitrogen source. The plates were incubated for 48 h at 37 °C.

To further investigate the potential role of XlnR in p*xylP* regulation, we aimed to identify *A. fumigatus* XlnR. Therefore, a blastp search (https://blast.ncbi.nlm.nih.gov/Blast.cgi) with *A. nidulans* XlnR (Q5AVS0, Tamayo et al., 2008) was conducted, which identified Afu2g15620 as the most likely *A. fumigatus* homologue (73% identity over a length of 962 amino acids). Vice versa, a blastp search using Afu2g15620 indicated that *A. nidulans* XlnR, *A. niger* XlnR (A2R5W7; 77% identity over a length of 896 amino acids; van Peij et al., 1998) and *Hypocrea jecurina* (anamorph *Trichoderma reesei*) XynR (XP_006966092; 52% identity over a length of 962 amino acids; Rauscher et al., 2006) as the proteins with the highest similarity. These data strongly suggested that Afu2g15620 is indeed the *A. fumigatus* XlnR homologue and the encoded protein gene was therefore termed XlnR. To confirm its function, we generated a respective gene deletion mutant (Δ*xlnR*) in *A. fumigatus* Afs77 (termed wt here) by replacement of the XlnR coding sequence with the *hph* selection marker gene. In line with *A. fumigatus* XlnR functioning as xylanolytic regulator, two independently generated Δ*xlnR* mutants displayed negligible growth on xylan, slightly reduced growth on xylose but wt-like growth on glucose and fructose as carbon source (Fig. 4). This growth pattern matches that of *A. nidulans* Δ*xlnR* mutants (Tamayo et al., 2008). This mutant now allowed to directly test its role in regulation of p*xylP* (see below).

### 3.4 Luciferase-mediated reporter assays elucidated the role of XlnR and in p*xylP* regulation

*In vivo* mVenus-mediated fluorescence measurements proved useful to analyze the p*xylP* activity under non-repressing/inducing conditions such as with 1%Fru/0.1%Xyl (Fig. 3). However, under conditions of low p*xylP* activity such as under repressing/inducing (1%Glc/0.1%Xyl) or repressing/non-inducing conditions (1%Glc), this methodology provided too low sensitivity, most likely due to the auto-fluorescence of hyphae. To increase sensitivity of the promoter analysis we tried luciferase instead of mVenus as reporter. Therefore, six p*xylP* versions including promoter truncations or mutations were fused to the codon optimized *Photinus pyralis* luciferase gene (GenBank accession numberKC677695) (Galiger et al., 2013) as reporter for promoter activity. All constructs were integrated in single copy at the *fcyB* locus as described above. For *in vivo* quantification of promoter activity, fungal strains were grown in minimal medium for 18 h at 37 °C and the promoter activity was determined as described in Materials and Methods.

**Fig. 5:**
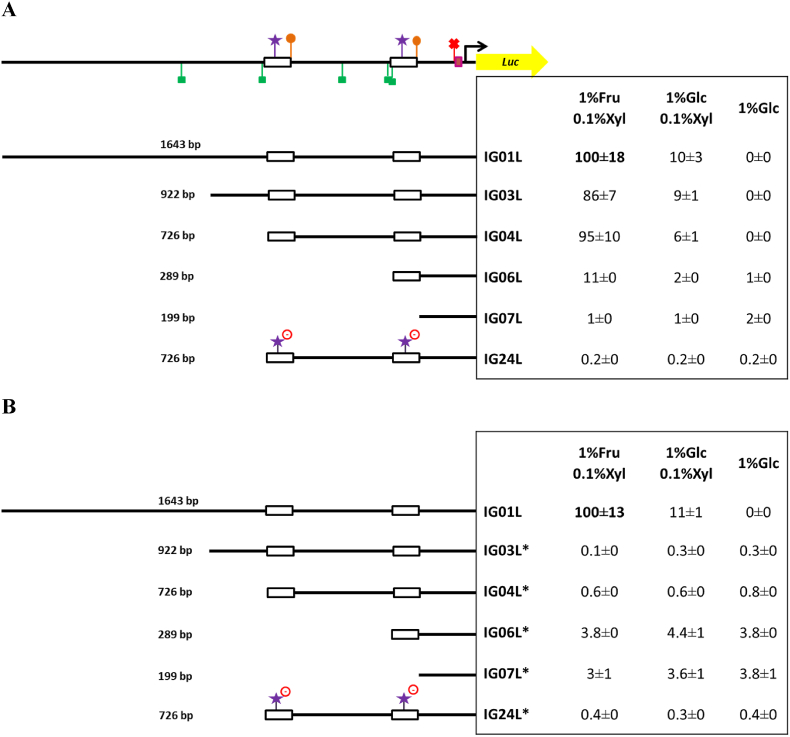
**Luciferase as reporter enabled to analyze effects of truncations and mutations on p*xylP* activity under conditions of low promoter activity in wt (A) and the Δ*xlnR* mutant strain (B).** Promoter activity was measured as described in Material and Methods. Shown values are the mean ± STD of three biological replicates normalized to IG01L grown with 1%Fru/0.1%Xyl for 18 h at 37 °C. Before normalization, background wt values were subtracted. Promoter activity of promoter versions marked with * in B was determined in the Δ*xlnR* mutant strain. Original data are shown in Supplementary Table S3A.

To simplify comparison, the nomenclature of the promoter versions was kept the same with L (for luciferase) instead of V (for Venus) as suffix; the activity of all promoter versions was normalized to that of IG01L (Fig. 5A), which contains the original 1643-bp p*xylP* promotor and corresponds to the IG01V-Venus reporter construct. As found above using mVenus as reporter (Fig. 3), the truncated promoter versions IG03L and IG04L retained high promoter activity with 1%Fru/0.1%Xyl, displayed significant activity with 1%Glc/0.1%Xyl and lacked activity with 1%Glc (Fig. 5A). Interestingly, IG04L showed slightly higher activity than IG03L with 1%Fru/0.1%Xyl (Fig. 5A), which contrasts the Venus-reporter data (Fig. 3), and the activity with 1%Glc/0.1%Xyl was about 3-fold higher compared to the Venus-reporter experiments, which underlines the higher sensitivity of the luciferase-reporter system. Similar to the Venus-reporter experiments (IG06V, Fig. 3), truncation of the region upstream of the proximal 91bpDS in IG06L caused significant reduction of promoter activity but retained slight xylose-inducibility (Fig. 5A). Interestingly, this promoter version showed low promoter activity (1.0%) also with 1%Glc. Further truncation to 199 bp in IG07L, which eliminates both 91bpDS, resulted in low (1–2%) inducer-independent constitutive promoter activity (Fig. 5A). Taken together, the promoter activities mediated by IG06L and IG07L compared to IG04L, indicate that the promoter region between −726 bp (IG04L) and −199 bp (IG07L) contains not only all the major regulatory elements required for inducer-mediated activation of p*xylP* but also mediate repression in the absence of the inducer. Notably, mutation of both putative XlnR-recognition motifs (5′-GGCTAAA-3′ to 5′-CATTAAA-3′) in the 91bpDS of IG04L, leading to IG24L, abrogated xylose-induced promoter activity and caused weak constitutive promoter activity (0.2–0.4%) even with 1%Glc (Fig. 5A). These data indicate that these motifs, and consequently most likely XlnR, are essential not only for xylose-induced promoter activation but are also involved in full repression of p*xylP* under repressing/non-inducing conditions. The about 5.5-fold reduced promoter activity of IG06L in 1%Glc/0.1%Xyl compared to 1% Fru/0.1%Xyl (Fig. 5A) indicates that glucose repression is still functional in the absence of all CreA motifs and suggests that glucose repression of *xylP* is at least partially indirect.

To investigate the role of XlnR in regulation of p*xylP*, promoter activity of five p*xylP* versions was analyzed in the Δ*xlnR* strain. Lack of XlnR resulted in weak and largely constitutive promoter activity of all five versions, IG03, IG04, IG06, IG07 and IG24 (Fig. 5B). In other words, lack of XlnR eliminated xylose-induced promoter activity in IG03L, IG04L and IG06L, which proves that XlnR is indeed responsible for xylose-induced promoter activation. Compared to wt (IG03L, IG04L; Fig. 5A), lack of XlnR slightly increased (0.3%–0.8%) promoter activity of IG03L, IG04L also in 1%Glc (Fig. 5B), which indicates that XlnR is also involved in repression in the absence of inducer. Similar to analysis in the wt (Fig. 5A), IG06L and IG07L showed higher promoter activity than IG03L and IG04L in 1%Glc in Δ*xlnR* (Fig. 5B). These data indicate that the region upstream of the proximal 91bpDS is involved also in XlnR-independent repression of p*xylP*. Promotor version IG24L displayed similar low and largely constitutive promoter activity in both wt (Fig. 5A) and Δ*xlnR* (Fig. 5B) underlining that XlnR operates via the mutated XlnR consensus sequences that are mutated in these promoter versions.

Taken together, luciferase as reporter provided higher sensitivity of the promoter studies compared to mVenus. The luciferase-mediated promoter studies demonstrated that XlnR is responsible for xylose-mediated induction of *xylP* via the identified consensus binding motifs present in the two 91bpDS, that XlnR might also act negatively under non-inducing conditions, and that the region upstream of the proximal 91bpDS (−286 bp, IG06) is not only important for inducer-mediated activation but also for XlnR-independent repression in the absence of inducer.

Due to the identified importance of the two 91bpDS in transcriptional control of *xylP*, we investigated the effect of insertion of a third 91bpDS upstream of upstream of pIG03L, resulting in pIG25L (Supplementary Figure S1B). This genetic engineering increased promoter activity particularly during low inducing/repressing conditions, e.g., 5-fold with 1%Glc/0.05%Xyl and 2.8-fold with 1%Glc/0.1%Xyl (Table 3). Moreover, this manipulation increased maximal promoter activity in 1%Fru/0.1%Xyl about 1.3-fold without impacting repression in 1%Glc (Table 3).

**Table 3:** Integration of a third 91bpDS in IG03L resulting in IG25L increases promoter activity during inducing/repressing and inducing non-repressing conditions. Promoter activity was measured as described in Material and Methods. The shown values are the mean ± STD of three biological replicates normalized to IG03L grown with 1%Fr/0.1%Xyl after growth for 18h at 37 °C. Original data are shown in Supplementary Table S4A.

	Carbon source						
	1%Glc	1%Glc 0.01%Xyl	1%Glc 0.02%Xyl	1%Glc 0.03%Xyl	1%Glc 0.05%Xyl	1%Glc 0.1%Xyl	1%Fru 0.1%Xyl
**IG3L**	0 ± 0	0 ± 0	1 ± 1	1 ± 1	2 ± 2	11 ± 2	**100 ± 10**
**IG25L**	0 ± 0	0 ± 0	1.4 ± 1	3 ± 1	10 ± 1	31 ± 3	129 ± 9

### 3.5 Comparing the pxyl*P* and *tet-On* promoter system

One of the most widely used conditional promoter is the tetracycline/doxycycline-induced TetOn system, here termed TetOn^*oliC*^ (using the *oliC* minimal promoter for driving expression of the target gene) (Helmschrott et al., 2013). So far, comparison of *xylP* and TetOn systems suffered of their use for driving expression of different target genes at different genomic loci. Here we compared the p*xylP* promoter with TetOn^*oliC*^ driving expression of the very same gene encoding luciferase integrated at the same genomic locus, the *fcyB* locus. Table 4 shows the mean of raw data ± STD of three biological replicates without normalization and without subtracting the wt background to better visualize the leakiness of the analyzed promoters. However, for calculating the fold-induction, the wt background was subtracted. p*xylP* showed about 2.6-fold higher basal promoter activity compared to wt background under repressing/non-inducing conditions (1%Glc) and about 2404-fold higher activity under non-repressing/inducing conditions (1%Fru/0.1%Xyl) compared to repressing/non-inducing conditions (1%Glc). TetOn^*oliC*^ displayed a basal promoter activity that was 8-fold higher than the wt background and about 52-fold induction with 10 μg/ml and 121-fold induction with 20 μg/ml doxycycline (Dox). These data demonstrate that compared to p*xylP*, TetOn^*oliC*^ displays an about 4.5-fold higher basal level and an about 4.4-fold lower maximal activity when comparing TetOn^*oliC*^ with 20 μg/ml doxycycline and p*xylP* in 1%Fru/0.1%Xyl. Taken together p*xylP* showed a higher maximal promoter activity, lower leakiness and consequently higher dynamic range than TetOn^*oliC*^.

**Table 4:** **Comparison of p*xylP* and TetOn**^***oliC***^**promoter activities.** Promoter activity was measured as described in Material and Methods. Shown values are the mean ± STD of three biological replicates after growth for 18 h at 37 °C. Original data are shown in Supplementary Table S4B.

	Carbon source		
	1%Glc	1%Glc 0.1%Xyl	1%Fru 0.1%Xyl
**IG01L**	46 ± 10	10550 ± 811	67358 ± 11051
**wt**	18 ± 4	20 ± 5	29 ± 6
	1%Glc	1%Glc 10 μg/ml Dox	1%Glc 20 μg/ml Dox
**TetOn**^***olic***^	144 ± 7	6616 ± 458	15351 ± 1270
**wt**	18 ± 4	11 ± 1	12 ± 3

### 3.6 Discussion

As summarized in the Introduction, p*xylP* from *Penicillium chrysogenum* was shown to mediate conditional gene expression in various mold species. Despite its intensive use in different laboratories, the essential regulatory elements in p*xylP* remained uncharacterized so far. In this study, two different reporters were used for monitoring p*xylP* activity. mVenus as reporter (Fig. 3) enabled easy *in vivo* quantification of high promoter activities, while firefly luciferase provided less background and was therefore better suited for analysis of low promoter activities (Fig. 5).

Mutational analysis using mVenus as reporter (Fig. 3) demonstrated the importance of the putative TATA-box, a pyrimidine-rich region located between the putative TATA box and the transcription start sites, both copies of the 91bpDS, as well as putative binding sites for the xylanolytic transcription factor XlnR, and a GATAA motif within the distal 91bpDS element. To further analyze the impact of XlnR, we generated an *A. fumigatus* mutant lacking XlnR, which displayed significantly decreased growth on xylan, slightly decreased growth on xylose but wt-like growth on glucose or fructose as carbon source (Fig. 4). These data demonstrated conservation of the role of XlnR in activation of xylan degradation and xylose utilization in *A. fumigatus* as shown previously in other fungal species (Klaubauf et al., 2014). In agreement with XlnR-dependence of p*xylP*, XlnR inactivation as well as mutation of both putative XlnR binding motifs abrogated xylose-induction of p*xylP* (Fig. 5). Interestingly, lack of XlnR as well as mutation of both putative XlnR increased basal p*xylP* activity, which indicates that XlnR might also contribute to repression under repressing/non-inducing conditions (Fig. 5). Repressing activity of XlnR has been predicted previously in *A. niger* (Hasper et al., 2004; Stricker et al., 2008). Elimination of both 91bpDS copies, which each comprise a single XlnR binding motif (Fig. 5) or mutation of both XlnR binding motifs completely abrogated inducer response of p*xylP* (Fig. 3, Fig. 5). In contrast, elimination of only a single XlnR binding motif by individual deletion of the 91bpD copies significantly decreased but did not completely abrogate the inducer response (Fig. 3, Fig. 5). These data indicate that a single XlnR motif mediates weak xylose induction but that full p*xylP* activity requires synergism of two XlnR motifs. In this respect, it is noteworthy that the XlnR homologue of *Hypocrea jecorina* is predicted to activate as a dimer binding to two recognition motifs (Stricker et al., 2008). The crucial role of the GATAA motif in the distal 91bpDS element indicates regulation by a GATA-type transcription factor. *Aspergillus* species possess six functionally characterized GATA-type transcription factors termed AreA, AreB, SreA, LreA, LreB and NsdD; which are involved in control of utilization of nitrogen sources, carbon metabolism, iron acquisition, light response as well as sexual and asexual development (Jiang et al., 2021). It remains to be shown if the identified GATAA motif is indeed recognized by a GATA-type transcription factor. Interesting to note, NsdD was implicated in regulation of production of cellulases and xylanases in *Penicillium oxalicum* (He et al., 2018), while AreA was shown to be involved in regulation of cellulases in *Trichoderma reesei* (Qian et al., 2019).

Remarkably, the 1579-bp p*xylP* was found to act bi-bidirectionally with a similar regulatory pattern by driving expression of the upstream-located arabinofuranosidase-encoding *axs5* gene (Sakamoto et al., 2011). In agreement with the presented reporter gene assays (Fig. 3), *axs5* was previously found to be induced by xylose via semiquantitative RT-PCR analysis of transcript levels. Notably, p*xylP* displayed lower activity into the *axs5* direction compared to the *xylP* direction. The co-regulation of *xylP* and *axs5* is meaningful as cooperation of the encoded enzymes is required for degradation of xylan. The higher expression of *xylP* compared to *axs5* might be physiologically relevant due to the higher abundance of xylose compared to arabinose in xylan (Rahman et al., 2003).

Truncation of p*xylP* to 199 bp demonstrated that the upstream region including the two 91bpDS copies mediates not only inducer-dependent activation but also XlnR-independent repression in the absence of inducer (Fig. 5).

The presence of glucose, but not of fructose was found to repress xylose-induction of p*xylP* activity. Due to the presence of five putative binding sites for CreA (Fig. 2), which mediates transcriptional downregulation of genes for the degradation of less-preferred sugars in the presence of favorable carbon sources such as glucose (Kowalczyk et al., 2014; Zadra et al., 2000) supposed that p*xylP* is subject to CreA-mediated carbon catabolite repression. Two lines of evidence, however, indicated that glucose mediated repression might not be CreA-dependent: (i) truncation of p*xylP* combined with deletion of all *in silico* predicted CreA motifs did not relieve glucose repression in 1%Glc/0.1%Xyl (Fig. 3, Fig. 5) and (ii) increasing the xylose concentration from 0.1% to 0.5 or 1% in the presence of 1%Glc increased p*xylP* activity approximately 8-fold and 12-fold, respectively (Table 2). Possibilities for glucose-repression independent of direct CreA regulation include (i) indirect CreA regulation via CreA transcriptional repression of the xylose-induced activator XlnR as indicated for the xylanase-encoding *xlnA* and *xlnB* genes in *A. nidulans* (Tamayo et al., 2008), (ii) inducer exclusion by CreA-mediated repression of xylose transporters as found for *A. nidulans* XtrD (Colabardini et al., 2014), (iii) inducer exclusion by competition of glucose and xylose for uptake by xylose transporters as all xylose transporters are competitively inhibited by glucose (Farwick et al., 2014) or (iv) a combination of the different possibilities.

Comparison with the widely used TetOn^*oliC*^ promoter (Helmschrott et al., 2013) demonstrated that p*xylP* shows a higher maximal promoter activity, lower basal activity under repressing/non-inducing conditions and consequently a higher dynamic range than TetOn^*oliC*^.

Characterization of functional elements followed by engineering of p*xylP* also opened new methodological opportunities for its application: (i) truncation of the originally used 1643-bp promoter fragment to 725 bp (IG04Vand IG04L, Fig. 3, Fig. 5) and further deletional mutagenesis to 543 bp (IG16V, Fig. 3) largely preserved the regulatory pattern, which facilitates cloning procedures; (ii) the 1579-bp p*xylP* fragment was found to act bi-bidirectionally with a similar regulatory pattern (IG15V, Fig. 3), which opens the possibility of bidirectional use of p*xylP* for conditional co-expression of two genes; (iii) fusion of a third 91bpDS element to the 925-bp promoter fragment significantly increased promoter activity particularly during low inducer availability under repressed conditions (IG25L, Fig. 5); this changed regulatory pattern might be particularly useful for *in vivo* studies in murine infection models or in the presence of repressing glucose; (iv) truncation to 199 bp rendered p*xylP* insensitive to xylose induction and slightly increased the basal activity, which qualifies this promoter version as a minimal promoter for functional studies of promoter elements.

Taken together, this study revealed insights into regulation of the xylanolytic system in *A. fumigatus*, identified several functional elements of p*xylP* and opened new methodological opportunities for its application.

## CRediT authorship contribution statement

**Annie Yap:** Investigation, Validation, Visualization, Methodology, Writing – original draft, Writing – review & editing. **Irene Glarcher:** Investigation, Validation, Visualization, Writing – original draft, Writing – review & editing. **Matthias Misslinger:** Investigation, Visualization, Writing – review & editing. **Hubertus Haas:** Conceptualization, Methodology, Supervision, Writing – original draft, Writing – review & editing, Funding acquisition.

## Declaration of competing interest

The authors declare that they have no known competing financial interests or personal relationships that could have appeared to influence the work reported in this paper.

## Data Availability

No data was used for the research described in the article.
