# Supplementary material for: Characterization and engineering of the xylose-inducible xylP promoter for use in mold fungal species
Source: Metab Eng Commun. 2022 Nov 19;15:e00214. doi: 10.1016/j.mec.2022.e00214 (PMC9703631; doi:10.1016/j.mec.2022.e00214)
Supplement: Multimedia component 1 [file mmc1.pdf]

Table S1: Oligonucleotides used in this study. Nucleotides in small letters are add-on sequences for cloning via NEB builder; nucleotides in small letters in italics represent inserted mutations.

Plasmid	Description	Primer Sequence
pIG01V	plasmide backbone	actcaagcttAAACAGCTATGACCATGATTAC
		gcatcagtgcTGTGAAATTGTTATCCGCTC
	p*xylP*	caatttcacaGCACTGATGCGAGCAACAG
		tgctgaccatGGTTGGTTCTTCGAGTCG
	mVenus	agaaccaaccATGGTCAGCAAGGGCGAG
		gctgccatggTTACTTGTACAGCTCGTCCATG
	trpC terminator	gtacaagtaaCCATGGCAGCAGTGATTTC
		atagctgtttAAGCTTGAGTGAGGGTTGAG
pIG02V	Site directed mutagenesis of pIG01V	TTCTGTTCTG*ggatcc*TATCGCCTCCATCCCTCCC
		GCCAGCATGGCATGTCTT
pIG03V	Site directed mutagenesis on pIG01V	ATAACTCATAGATGTCTCG
		TGTGAAATTGTTATCCGC
pIG04V	Site directed mutagenesis on pIG01V	CAGACTAAAGTCTTCTATAGG
pIG05V	Site directed mutagenesis on pIG01V	GGCAAGCATCCGTTCCCC
		TGTGAAATTGTTATCCGCTCACAATTC
pIG06V	Site directed mutagenesis on pIG01V	CAGAATAAAGTCCTCTCTG
pIG13V	Site directed mutagenesis on pIG01V	tgatagagaACGATGTCTTCTATCACAC
		ctgatagggaTACTTATACAGAACAGAAGC
pIG15V	pIG01V backbone	cttgacgacaATGGTCAGCAAGGGCGAG
		agaaccaaccTGTGAAATTGTTATCCGCTCACAATTC
	p*xylP* reverse complementary	caatttcacaGGTTGGTTCTTCGAGTCG
		tgctgaccatTGTCGTCAAGATTGTAGTG
pIG16V	Site directed mutagenesis on pIG01V	AATAAAGTCCTCTCTGCG
		ATGACGTCTATCACCTCC
pIG17V	Site directed mutagenesis on pIG01V	AGCATTTA*cat*TAAAAAAATGATGGAAC
		CCTATAGAAGACTTTAGTCTGTGTG
pIG19V	Site directed mutagenesis on pIG01V	CAGAATAAAGTCCTCTCTG
		TATTTTATCCAAGATCCTAGT
pIG20V	Site directed mutagenesis on pIG01V	TGTAAATTCGTTCTCCATTCGTGAAC
		GAGTGCATGTGGGGCACG
pIG21V	Site directed mutagenesis on pIG01V	AAAATGATGGAACATTATTTCATC

pIG22V	Site directed mutagenesis on pIG01V	GGCAAGCATCCGTTCCC<br>ATTTTTTTAGCCTAAATGCTCCTATAGAAGAC
pIG23V	Site directed mutagenesis on pIG01V	TATTTTCGACAAGATCCTAGTAAATATTTAGGATAAAT
pIG01L	plasmide backbone	AAACAGCTATGACCATGATTAC<br>TGTGAAATTGTTATCCGCTC
	p*xylP*	agcggataacaatttcacaGCACTGATGCGAGCAACAG<br>atgttcttggcgtcctccatGGTTGGTTCTTCGAGTCG
	luc	catcgactcgaagaaccaaccATGGAGGACGCCAAGAAC<br>gaaatcactgctgccatggCTAGACGGCGATCTTGCCGCC
	trpC terminator	ggcggcaagatcgccgtctagCCATGGCAGCAGTGATTTC<br>aatcatggtcatagctgtttAAGCTTGAGTGAGGGTTG
pIG07L	Site directed mutagenesis on pIG01L	TGTAAATTCGTTCTCCATTCG
pIG24L		GCGAGAATTTA*cat*TAAAGAAAGATC<br>GATCTTTCTTTAATGTAAATTCTCGC
Δ*xlnR*	5´NCR	GCCCTTCTGGCCAGGGTG<br>tagttctgttaccgagccggCCTCACGGGTGAAGCAGAGC
	3´NCR	gctctgaacgatatgctcccGATTGAGCTCGTGTTTCG<br>CCGTTTCTTTGCTGCGTCC
	hph	ccggctcggtaacagaactaACGGCGTAACCAAAAGTCAC<br>gggagcatatcgttcagagcTCTTGACGACCGTTGATCTG
	fusion cassette	TTCACTTCAGAATTCCCCTCGC<br>TACGGGAGACAAAGGCGAG
TetOn	plasmide backbone	AAACAGCTATGACCATGATTAC<br>TGTGAAATTGTTATCCGCTC
	TetOn^{oliC}	atcacacggcctgagtggccATGGAGGACGCCAAGAACAT<br>atgttcttggcgtcctccatGGCCACTCAGGCCGTGTGAT
	luc	atcacacggcctgagtggccATGGAGGACGCCAAGAACAT<br>ccgcttgagcagacatcaccATGGAGGACGCCAAGAACAT<br>gaaatcactgctgccatggCTAGACGGCGATCTTGCCGCC
	trpC terminator	ggcggcaagatcgccgtctagCCATGGCAGCAGTGATTTC

Table S2. **Table showing the raw fluorescence values of three biological replicates from one transformant**.
(A) Raw values of mVenus reporter assays shown in Table 2. (B) Raw values of mVenus reporter assays shown in Figure 3.

A

		1%Xyl	1%Glc 0%Xyl	1%Glc 0.1%Xyl	1%Glc 0.5%Xyl	1%Glc 1%Xyl	1%Fru 0%Xyl	1%Fru 0.1%Xyl
IG01V	1	37585	878	1665	9707	14130	844	37446
	2	42973	839	1680	9206	14446	845	38583
	3	44050	873	1678	10008	14390	864	37097
wt	1	839	852	851	899	911	801	844
	2	845	894	862	855	954	811	877
	3	840	911	825	1001	977	821	857

B

	1%Fru 0.1%Xyl			1%Glc 0.1%Xyl		
	1	2	3	1	2	3
IG01V	29054	28733	28293	1678	1598	1605
IG02V	3262	3205	3049	1006	959	969
IG03V	24766	23554	22991	1508	1526	1511
IG04V	25003	25484	25528	1263	1268	1283
IG05V	2535	2455	2484	929	943	955
IG06V	2301	2277	2200	1067	1089	1057
IG07V	29942	28047	28893	1722	1711	1727
IG13V	7015	7093	6858	1061	1096	1093
IG15V	14226	13865	12931	1256	1220	1237
IG16V	27021	26547	25632	1720	1702	1724
IG17V	4182	4077	4172	949	934	938
IG18V	7857	7500	7461	1102	1114	1111
IG19V	849	819	829	872	823	863
IG20V	1532	1511	1442	972	955	995
IG21V	4419	4209	4142	901	907	897
IG22V	11593	11050	11610	894	862	883
IG23V	18761	17182	17367	1012	1082	1020
wt	939	945	940	952	994	986

Table S3. Table showing the raw bioluminescence values of three biological replicates from one transformant. (A) Raw values of the luciferase reporter assays shown in Figure 5A. (B) Raw values of the luciferase reporter assays shown in Figure 5B.

A

	1%Glc			1% Glc 0.1% Xyl			1% Frau 0.1% Xyl		
	1	2	3	1	2	3	1	2	3
IG01L	71	76	94	29483	18180	23881	270637	229934	189231
IG03L	130	97	129	23518	19031	17379	217046	187671	187358
IG04L	107	174	176	14903	15133	12311	242450	194827	218638
IG06L	3127	2736	2862	5055	5640	5911	25667	25198	23684
IG07L	4833	3159	3266	3024	3177	3858	3111	3220	2569
IG24L	316	546	411	600	451	698	556	789	873
wt	18	23	39	72	70	99	392	350	285

B

	1%Glc			1%Glc 0.1%Xyl			1%Fru 0.1%Xyl		
	1	2	3	1	2	3	1	2	3
IG03L*	414	366	410	322	303	559	470	490	307
IG04L*	881	1213	675	681	963	703	1318	912	1037
IG06L*	4935	4232	4481	5911	4538	5155	5091	5259	4874
IG07L*	3577	5158	4729	4070	5237	3551	4572	3579	3379
IG024L*	492	429	448	409	412	387	489	487	546
wt	26	23	20	57	60	70	319	349	262
IG01L	72	62	83	13014	11702	14491	131233	131553	104809

Table S4. Table showing the raw bioluminescence values of three biological replicates from one transformant. (A) Raw values of the luciferase reporter assays shown in Table 3. (B) Raw values of the luciferase reporter assays shown in Table 4.

A

	1%Glc			1%Glc 0.01%Xyl			1%Glc 0.02%Xyl			1%Glc 0.03%Xyl		
	1	2	3	1	2	3	1	2	3	1	2	3
IG25L	70	61	71	440	1391	1107	5212	3489	1619	10697	8707	6167
IG3L	87	65	58	197	244	429	1469	1198	1846	5167	4633	4706
wt	59	67	58	171	276	317	476	502	596	885	861	699
	1%Glc 0.05%Xyl			1%Glc 0.1%Xyl			1%Fru 0.1%Xyl					
	1	2	3	1	2	3	1	2	3			
IG25L	26414	18692	27279	71066	85803	78798	346319	320536	300154			
IG3L	3039	3524	3592	31076	27174	20314	300281	232600	267174			
wt	45	42	50	108	107	221	539	522	427			

B

	1%Glc			1%Glc 0.1%Xyl			1%Fru 0.1%Xyl		
	1	2	3	1	2	3	1	2	3
IG01L	44	38	57	10142	10024	11484	79897	63135	59041
wt	14	18	21	15	25	19	24	36	28

	1%Glc			1%Glc 10µg/ml Dox			1%Glc 20µg/ml Dox		
	1	2	3	1	2	3	1	2	3
TetOn*oliC*	148	136	147	6889	6872	6087	16775	14336	14941
wt	14	18	21	11	12	11	9	10	16

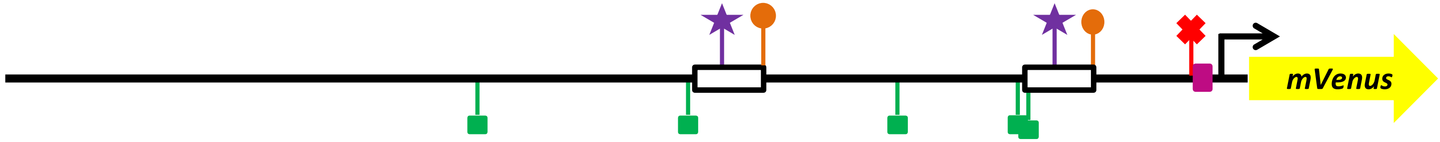

A ✖ TATAA motif

wt: 5´-TGTATAAGTA

IG02V: 5´-TGGGATCCTA

■ Pyrimidine-rich sequence

wt: 5´-TATCGCCTCCATCCCTCCCCGAC

IG13V: 5´-TATCCCTATCAGTGATAGAGAAC

★ XlnR motif

wt: 5´-TAGGCTAAAAA

IG17V: 5´-TACATTAAATA

● GATAA motif

wt: 5´-TGGATAAAA

IG23V: 5´-TGTCGAAAA

▭ Distal 91bpDS truncation

wt: 5´-CAGACTAAAGTCTTCTATAGGAGCATTTAGGCTAAAAAAATGATGGAACATTATTTCATCCTAAATATTTACTAGGATCTTGGATAAAATA

IG21V:5´-------------------------------------AAAATGATGGAACATTATTTCATCCTAAATATTTACTAGGATCTTGGATAAAATA

IG22V:5´-CAGACTAAAGTCTTCTATAGGAGCATTTAGGCTAAAAAAAT--

Proximal 91bpDS truncation ▭

wt: 5´-CAGAATAAAGTCCTCTCTGCGAGAATTTAGGCTAAAGAAAGATCGACTATAATTTCATCCGAGCTATTTGTTAGGATTTTGGATAAAATA

IG20V:5´---

B IG25L:5´-CAGATTAAAGTCATCTTTCAGAGGATTTAGGCTAAACAAAGATAGATGATCATTTCATCCTATTTATTTCGTAGGATGTTGGATAAAATA

Figure S1. Analyzed mutations and deletions in p*xylP*. (A) Putative binding motifs for transcription factors : TATAA in red, GATAA in orange, XlnR (5'-GGCTAAA) in purple and pyrimidine-rich sequence in pink. Mutations are in blue and deletions are marked by blue dashes. (B) Sequence of the third integrated 91bpDS synthetic DNA fragment with 70% similarity to the other two 91bpDS (for cloning reasons, we used the 5´-extension 5-GTGCCCCACATTGTATC-containing fragment. The exchanged, non-conserved nucleotides are shown in blue.

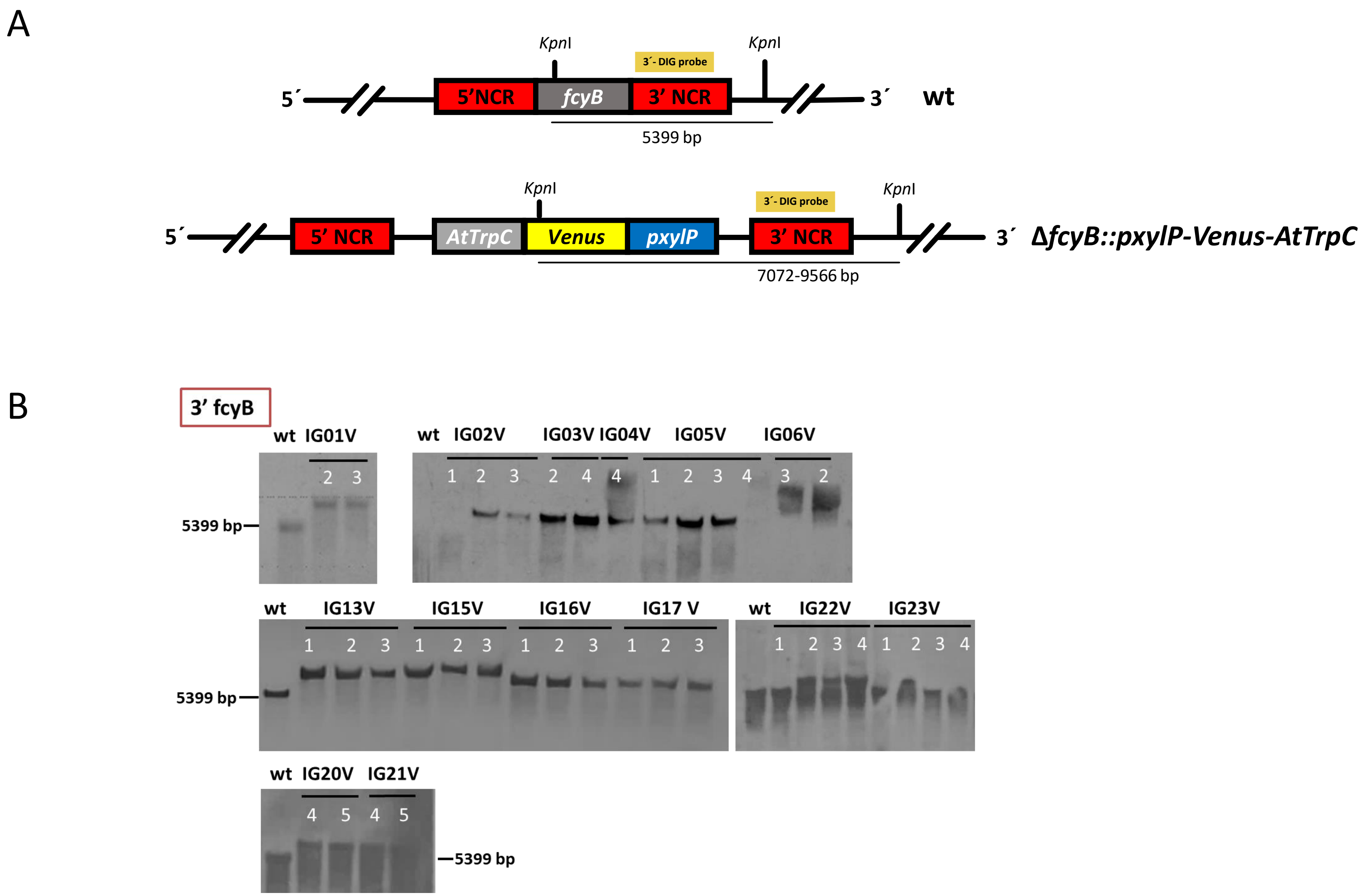

Figure S2. **Scheme for the genomic integration of the *pxylP* mVenus reporter constructs at the *fcyB* locus**. (A) Genomic organization of the *fcyB* locus in wt (wild-type) and Δ*fcyB.* Generated gDNA digestion with *Kpn*I resulted in a 5399-bp fragment for wt p*xylP* and fragments ranging from 7072-9566 bp for the different p*xylP* versions. (B) Southern blot analysis using respective DIG hybridization probes confirmed genetic manipulations.

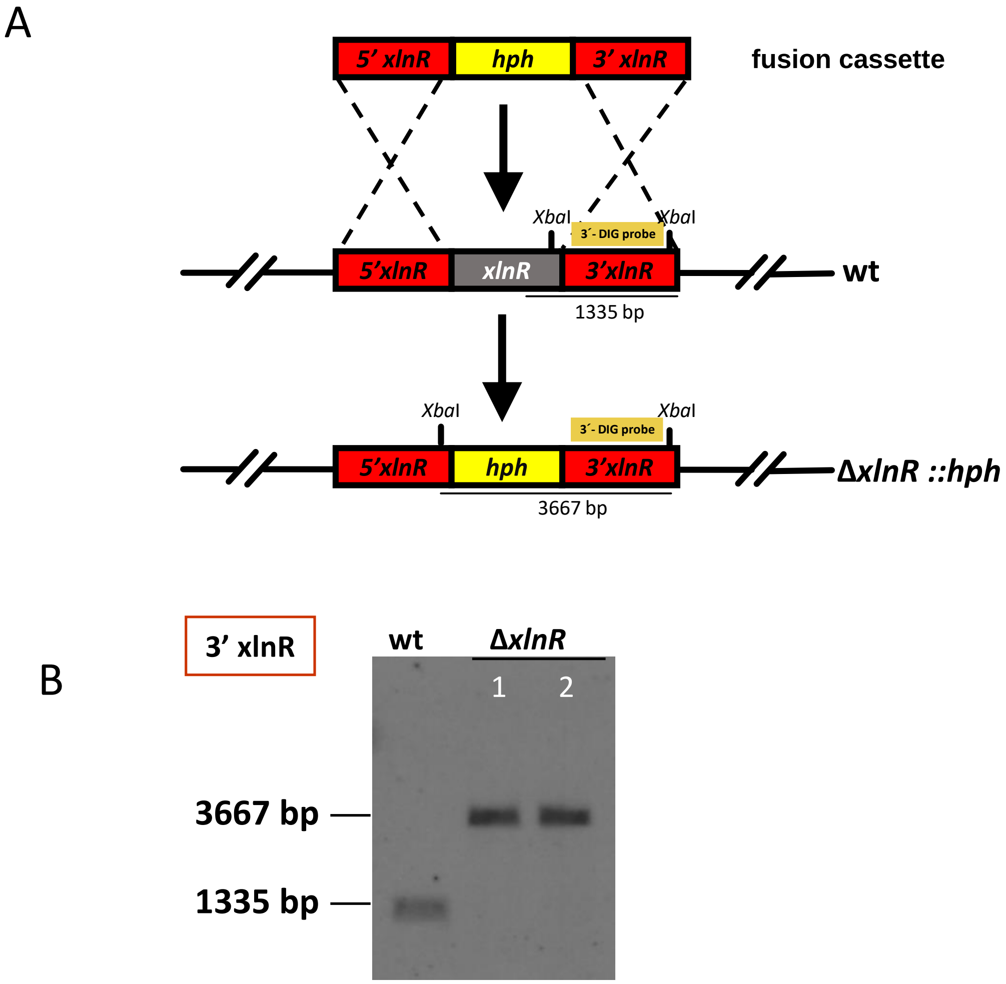

Figure S3. **Scheme for *xlnR* deletion in *A. fumigatus***. (A) Genomic organization of the *xlnR* locus in wt and Δ*xlnR*. Generated gDNA digestion with *Xba*I resulted in a 1355-bp and 3667-bp fragments for wt and Δ*xlnR*, respectively. (B) Southern blot analysis using respective DIG hybridization probe confirmed genetic manipulation.

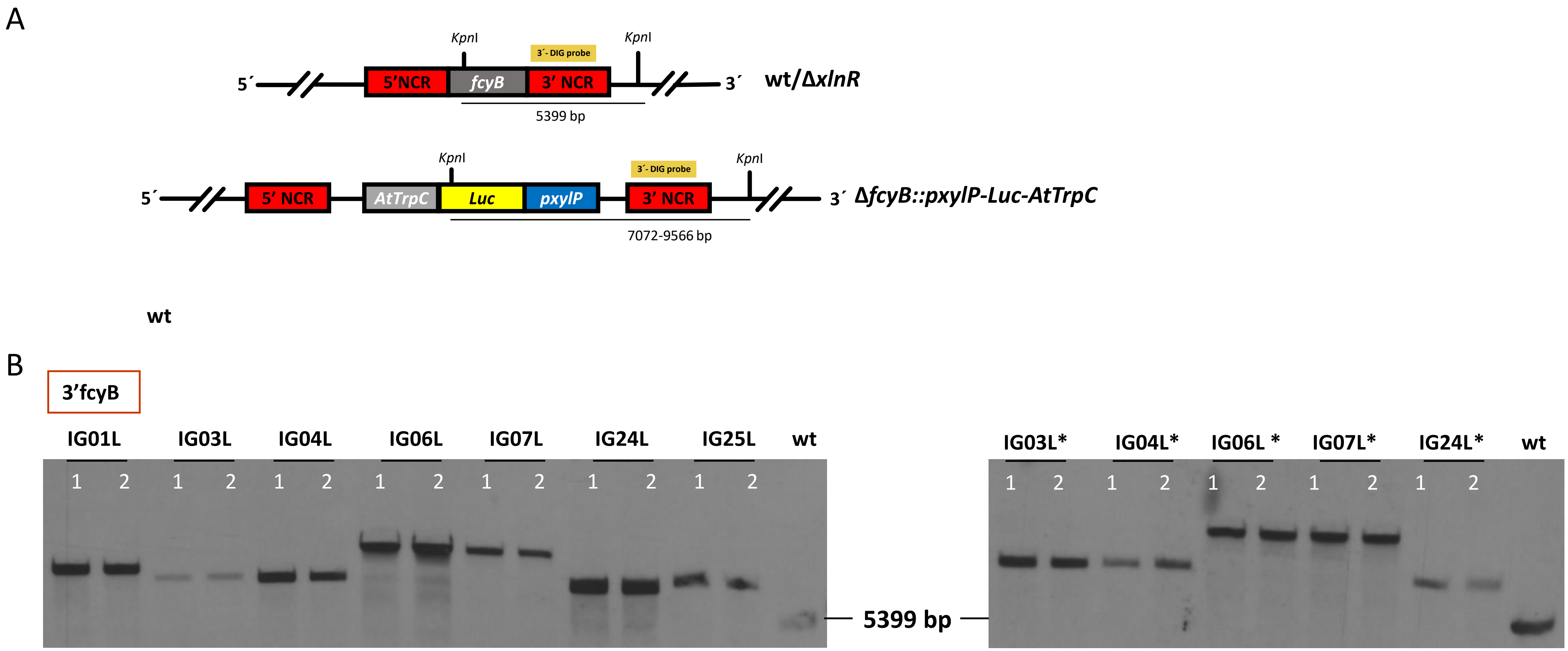

Figure S4. **Scheme for the genomic integration of p*xlyP* versions controlling luciferase-encoding gene at the *fcyB* locus**. (A) Genomic organization of the *fcyB* locus in wt and Δ*fcyB.* Generated gDNA digestion with *Kpn*I resulted in a 5399-bp fragment for wt/Δ*xlnR* and fragments ranging from 7072-9566 bp for the different p*xylP* versions. (B) Southern blot analysis using respective DIG hybridization probe confirmed genetic manipulation.

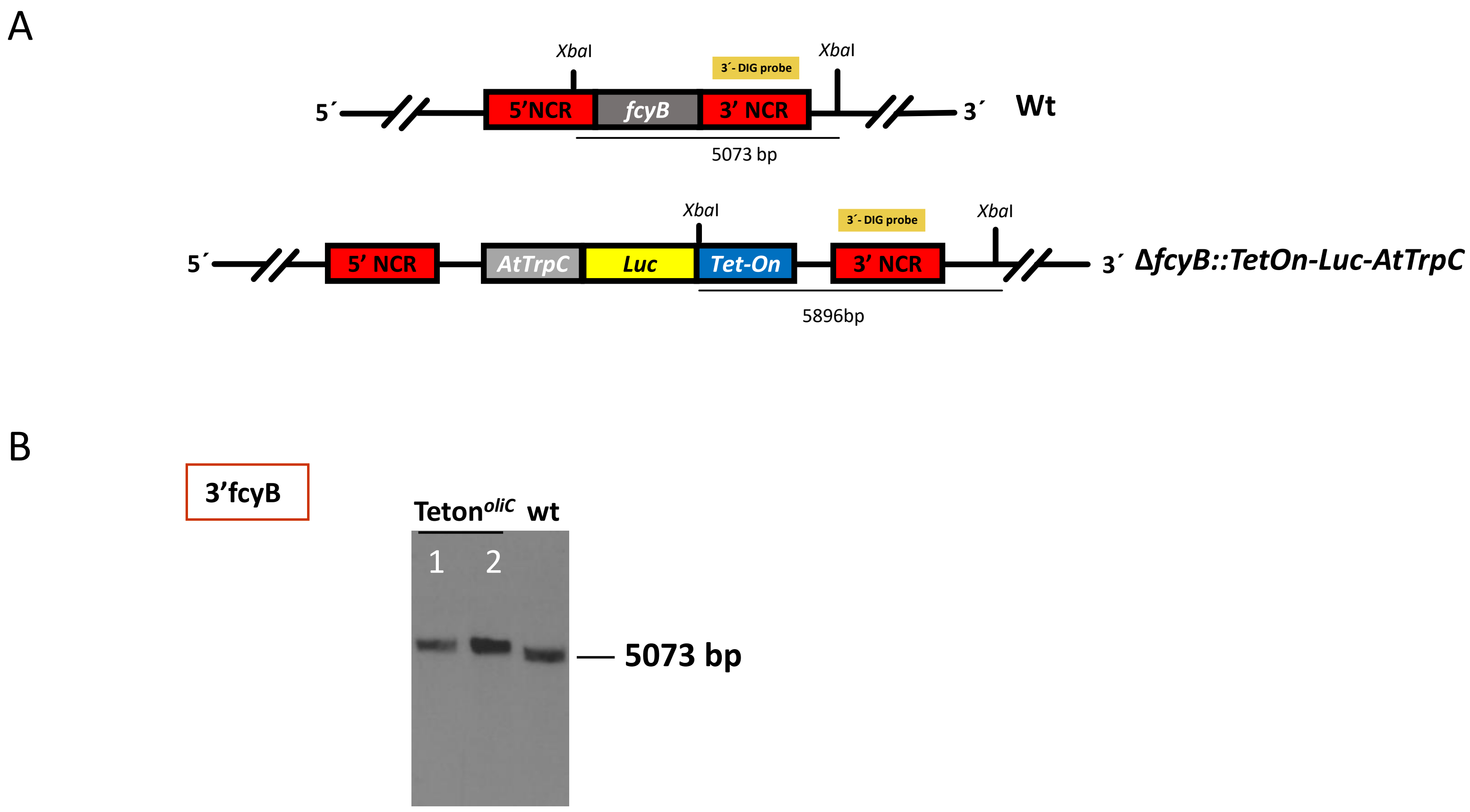

Figure S5. Scheme for genetic integration of the TetOn firefly luciferase reporter constructs at the *fcyB* locus. (A) Genomic organization of the *fcyB* locus in wt and Δ*fcyB.* Generated gDNA digestion with *Xba*I resulted in a 5073-bp fragment for wt and fragments ranging from 5896 bp for the different p*xylP* versions. (B) Southern blot analysis using respective DIG hybridization probe confirmed genetic manipulation.
